# Estimate of the global burden of cervical adenocarcinoma and potential impact of prophylactic human papillomavirus vaccination

**DOI:** 10.1186/1471-2407-13-553

**Published:** 2013-11-21

**Authors:** Jeanne M Pimenta, Claudia Galindo, David Jenkins, Sylvia M Taylor

**Affiliations:** 1 Worldwide Epidemiology, GlaxoSmithKline R&D, Stockley Park, Middlesex, UK; 2 Epidemiology, North American Vaccine Development, GlaxoSmithKline Vaccines, King of Prussia, North America, PA, USA; 3 DDL Diagnostic Laboratory, Rijswijk, The Netherlands; 4 GlaxoSmithKline Vaccines, Avenue Fleming 20, B-1300, Wavre, Belgium

**Keywords:** Uterine cervical neoplasm, Adenocarcinoma, Papillomavirus infections, Human papillomavirus vaccines

## Abstract

**Background:**

Data on the current burden of adenocarcinoma (ADC) and histology-specific human papillomavirus (HPV) type distribution are relevant to predict the future impact of prophylactic HPV vaccines.

**Methods:**

We estimate the proportion of ADC in invasive cervical cancer, the global number of cases of cervical ADC in 2015, the effect of cervical screening on ADC, the number of ADC cases attributable to high-risk HPV types -16, -18, -45, -31 and -33, and the potential impact of HPV vaccination using a variety of data sources including: GLOBOCAN 2008, Cancer Incidence in Five Continents (CI5) Volume IX, cervical screening data from the World Health Organization/Institut Català d'Oncologia Information Centre on HPV and cervical cancer, and published literature.

**Results:**

ADC represents 9.4% of all ICC although its contribution varies greatly by country and region. The global crude incidence rate of cervical ADC in 2015 is estimated at 1.6 cases per 100,000 women, and the projected worldwide incidence of ADC in 2015 is 56,805 new cases. Current detection rates for HPV DNA in cervical ADC tend to range around 80–85%; the lower HPV detection rates in cervical ADC versus squamous cell carcinoma may be due to technical artefacts or to misdiagnosis of endometrial carcinoma as cervical ADC. Published data indicate that the five most common HPV types found in cervical ADC are HPV-16 (41.6%), -18 (38.7%), -45 (7.0%), -31 (2.2%) and -33 (2.1%), together comprising 92% of all HPV positive cases. Future projections using 2015 data, assuming 100% vaccine coverage and a true HPV causal relation of 100%, suggest that vaccines providing protection against HPV-16/18 may theoretically prevent 79% of new HPV-related ADC cases (44,702 cases annually) and vaccines additionally providing cross-protection against HPV-31/33/45 may prevent 89% of new HPV-related ADC cases (50,769 cases annually).

**Conclusions:**

It is predicted that the currently available HPV vaccines will be highly effective in preventing HPV-related cervical ADC.

## Background

Invasive cervical cancer (ICC) is one of the leading causes of cancer in women and according to global estimates from 2008, there are approximately 530,000 new cases and 275,000 deaths annually [1]. Cervical cancer incidence and mortality has been reduced substantially in countries that have well-developed cervical screening programs [2]. This decline in incidence is mainly due to the increased detection and effective treatment of early precursors of squamous cell carcinoma (SCC), which is the most common histologic variant of cervical cancer. However, the effectiveness of screening in reducing the incidence of cervical adenocarcinoma (ADC) (including adenosquamous carcinoma [ASC]) is less clear, with many studies indicating that the relative and absolute incidence of ADC has actually increased, particularly among younger women [3-10]. In Western countries, with established cervical screening programs, ADC may represent up to 25% of ICC cases [4,11].

The natural history of cervical ADC is very different from that of SCC and this may explain the lower rates of detection of premalignancies during cytologic screening. The earliest precursor lesions of ADC are more difficult to define than those of SCC; lesions are more diverse, and invasive ADC is thought to develop particularly from a small focus of adenocarcinoma *in situ* (AIS) [7,11,12]. AIS is more difficult to sample than squamous precancer as it typically occurs within the endocervical canal and the cytological and curettage samples obtained can be difficult to diagnose accurately and reproducibly [11-13]. As a result of these factors, ADC is often diagnosed at a more advanced disease stage than SCC, and is generally associated with a worse prognosis [14-19].

Two prophylactic human papillomavirus (HPV) vaccines for the prevention of cervical cancer are now available and licensed in more than 100 countries. In clinical trials, these vaccines have shown good efficacy against high grade cervical lesions associated with HPV-16 and/or -18 [20-22]. The HPV-16/18 AS04-adjuvanted vaccine (*Cervarix*®) has also shown significant type-specific cross-protection for several non-vaccine oncogenic HPV types, including HPV-31, -33, -45 and -51, using both virological and histopathological endpoints [20,23]. For the quadrivalent HPV-6/11/16/18 vaccine (*Gardasil*®), significant cross-protection has also been reported for HPV-31, but not for other oncogenic HPV types to date [24].

However, no data are currently available regarding the efficacy of these vaccines specifically against ADC. Although AIS lesions were included within the definition of high-grade lesions in clinical trials evaluating the efficacy of these vaccines, the number of such lesions was relatively small [20,25]. Therefore, data on the current burden of ADC and histology-specific HPV type distribution are relevant to predict the future impact of such vaccines on ADC.

In this article, we estimate the global burden of disease due to ADC, investigate the effect of cervical screening on ADC, summarize data on HPV type-distribution in ADC and estimate the potential impact of HPV vaccination on ADC.

## Methods

### Predicted global burden of disease due to ADC in 2015

The projected number of ADC cases in 2015 was estimated using data from a number of published sources. Firstly, estimates of ICC cases by country were downloaded from GLOBOCAN 2008 [1]. Secondly, the proportion of ICC cases that were histologically confirmed ADC cases was extracted from previously published registry-specific data from the International Agency for Research on Cancer (IARC) Cancer Incidence for Five Continents (CI5) volume IX [26]. The IARC CI5 data, generally from 1998–2002, are presented by cancer registry and the majority of countries have multiple cancer registries. Where data from multiple registries per country were available, weighted averages were calculated to derive country-specific estimates for the proportion of ADC cases. Country-level proportions were derived from these weighted averages, and these data were applied to country-specific ICC cases derived from GLOBOCAN. Fewer countries are reported in CI5 than in GLOBOCAN 2008 (60 and 182, respectively); thus, countries were categorized into regions and sub-regions. If country specific data were not available, other countries within sub-regions were used to create a weighted average for that sub-region and these data were applied to all countries in that sub-region. For example, in the Eastern African sub-region, data were available for Uganda and Zimbabwe; these were used to create an Eastern African sub-regional weighted average which was applied to other countries in the Eastern African sub-region (e.g., Burundi, Comoros and Djibouti). Finally, data were then summed to estimate the total of ADC cases globally in 2015. Crude global incidence rates for ICC and ADC were calculated from the total number of cancer cases and the global female population estimate in 2015 (medium variant) from the United Nations world population prospects database [27].

In IARC CI5, ASC was categorized as “other” specified carcinoma rather than ADC, therefore, it was not possible to estimate the global burden of disease due to ADC including ASC [26].

### Country and regional age-standardized incidence rates of ADC

Registry-specific age-standardized incidence rates (ASIRs) of ADC are now published by the IARC in CI5 volume IX [26]. Weighted averages (using the total number of cases per registry to derive weights) were calculated to derive country-specific estimates where data on multiple registries per country were available. Regional weighted estimates of incidence were then calculated from country-level data by combining countries within regions as categorized by IARC.

### Effect of cervical screening on ADC

Data on screening coverage rates from cervical screening programs collated by World Health Organization/Institut Català d'Oncologia (WHO/ICO) Information Centre on HPV and Cervical Cancer were used to investigate whether there was an effect of cervical screening on the proportion of ADC among ICC cases [28] by country using linear regression.

### Type distribution of HPV in ADC

We summarized the global HPV type distribution in cervical ADC using data from two published sources [29,30]. The first published source was a meta-analysis, performed by Li and colleagues [29], of HPV type-specific prevalence from studies published between 1990 to 2010, including a total of 243 studies and 30,848 cases of ICC (3,538 cases of ADC or ASC). Contributing studies had wide geographic representation (86 countries), used a variety of PCR-based technology to detect HPV DNA, and samples came from a number of sources (fresh or fixed biopsies, or exfoliated cervical cells). Crude type-specific prevalence for 23 HPV types in the high-risk clade (HPV-16, -18, -26, -30, -31, -33, -34, -35, -39, -45, -51, -52, -53, -56, -58, -59, -66, -67, -68, -69, -70, -73, -82 and -85) [31] and low-risk types HPV-6 and -11 were presented as a proportion of all cases tested, unadjusted for the impact of multiple types.

The second source was a retrospective cross-sectional study, performed by de Sanjosé and colleagues, of 10,575 cases of ICC (951 cases of ADC or ASC) paraffin-embedded tissue blocks collected between 1949 and 2009 from worldwide historic archives in 38 countries [30]. Although the sample size and geographic representation is smaller than in the meta-analysis conducted by Li and colleagues [29], data were included because a common protocol was used for collection of specimens, histological confirmation and classification, and HPV testing (SPF_10_ broad-spectrum primers [SPF10-DEIA/LiPA25-polymerase chain reaction (PCR) system (SPF10-LiPA25; version 1, Labo Biomedical Products, Rijswijk, The Netherlands, based on licensed Innogenetics technology] followed by DNA enzyme immunoassay, genotyping with a reverse hybridization line probe assay and sequencing when required). HPV prevalence data (19 high-risk and 8 low-risk types) were reported for those cases which were positive for HPV DNA, and data analyses included algorithms of multiple infections to estimate type-specific relative contributions.

Crude data from Li and colleagues were adjusted to facilitate comparison across the two data sources. Firstly, data were adjusted so that each HPV type-specific prevalence was expressed as a proportion of HPV positive cases, i.e., each crude estimate was multiplied by 100/82 (as the overall HPV prevalence from this data source was 82% [29]). Secondly, because women infected with multiple types contribute several times in the numerator but only once in the denominator, the addition of the total percentages exceeded 100%; therefore we also adjusted the estimates of prevalence to sum to 100% (i.e., the total percentage of all HPV types was 110.7%, so each estimate of HPV type prevalence was multiplied by 100/110.7). Several studies have established, by laser capture microscopy and sensitive PCR, that only one HPV type is found in cancer cells or in a defined area of precancer even if a whole tissue specimen is positive for multiple HPV types. Such precise allocation has not been done in the epidemiological studies reviewed here, and allocation to an individual HPV type in multiple infections is usually based on its frequency in single infections. It is unclear whether this introduces errors into assessing the causal role of HPV types detected infrequently and as multiple infections [32,33].

Finally, to consolidate the HPV-type specific estimates from these two sources [29,30] into a single estimate for each type, we took a weighted average based on the total number of cases of ICC or ADC/ASC in each study.

### Potential impact of HPV vaccination

Vaccine efficacies against HPV types -16, -18, -45, -31 and -33 for the two currently available licensed HPV vaccines (*Gardasil*® and *Cervarix*®) were sourced from published data from double-blind, randomized, controlled, Phase III clinical studies [20,21,23]. In our calculations we used the point estimate and upper and lower limits of the confidence intervals for vaccine efficacy against high-grade cervical lesions (cervical intraepithelial neoplasia grade 2 or higher) associated with each HPV type. 96.1% and 95.89% confidence intervals were used for estimates of HPV-16/18 associated vaccine efficacy for *Cervarix*® and *Gardasil*®, respectively, due to adjustments for multiplicity. 95% confidence intervals were used for estimates of vaccine efficacy against other HPV types. Estimates of efficacy from these published sources were as follows: 98% (86 to 100%) associated with HPV-16 and -18 [20,21], 100% (42 to 100%) against HPV-45 [23], 89% (66 to 98%) against HPV-31 [23], and 82% (53 to 95%) against HPV-33 [23].

Using the calculated estimates for global burden of ADC, SCC and ICC in 2015 and the weighted averages of HPV type distributions [29,30], vaccine efficacy estimates were then used to calculate the proportion and number of new cases of ADC, SCC and ICC which could theoretically be prevented by HPV vaccination. We assumed 100% vaccine coverage in these calculations. We compared this to SCC, where the proportion of SCC within total ICC cases was calculated in the same manner as described for ADC.

## Results

### Global burden of disease due to ADC

**Figure 1:**
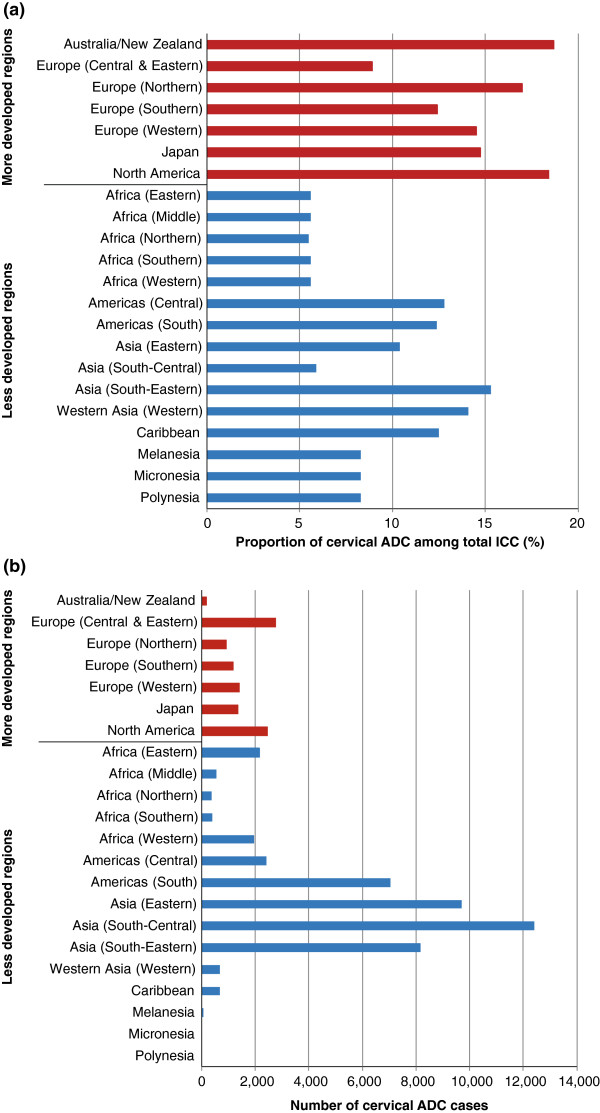
**Estimated proportion and number of new cervical ADC cases predicted in 2015 by region.** Panel **(a)** shows estimated proportion of new cervical ADC cases among total invasive cervical cancer (ICC) cases predicted in 2015 by region. Panel **(b)** shows estimated number of new cervical ADC cases predicted in 2015 by region.

The estimated global crude incidence rates of ICC and ADC in 2015 are 16.8 cases and 1.6 cases, respectively, per 100,000 women. IARC predicts the worldwide incidence of ICC in 2015 to be 607,402 cases. It was estimated that ADC represented 9.4% of all cases of ICC, giving a predicted worldwide incidence in 2015 of 56,805 cases. The estimated proportion of ADC among ICC cases by region with available data is shown in Figure 1a; this varied from 5.5% in the North African region to 18.7% in Australia/New Zealand. The majority of the regions classed as more developed by IARC had a relatively high proportion of ADC cases (generally >14%), whereas this was more variable among the least developed regions. The highest burden in terms of numbers of ADC cases in 2015 was centred in the least developed regions with over half (53%) of the total ADC cases seen in South and Eastern Asia (Figure 1b).

### Age-standardized ADC incidence rates

Country-level ASIRs [26] of ADC were very variable across countries (Table 1), with incidence per 100,000 women ranging from 0.2 in Algeria (Sétif) and Tunisia (Sousse) to 3.2 in Peru (Trujillo) and peaking at 3.4 in Thailand (3 registries). Within countries with multiple registries, there was also variability between registries (Table 1). For example, in China, there was an 8-fold difference in the minimum and maximum ASIR across 5 registries (ranging from 0.2 to 1.6 per 100,000 respectively) and similarly in Italy, a 5-fold difference was seen across 22 registries (ranging from 0.3 to 1.5 per 100,000 women). Conversely, other countries were more homogeneous and there was no or little difference in ASIR: Austria [2 registries] no difference; Turkey [2 registries] and Kuwait [2 registries] with difference of 0.1 per 100,000 women. The highest registry-specific ASIRs were seen from the Brasilia registry in Brazil (4.5 per 100,000 women) and Lampang in Thailand (3.9 per 100,000 women).

**Table 1:** Age-standardized incidence of cervical ADC by country

**Country, City (Number of registries)**	**Age-standardized incidence rate (ASIR) per 100,000 women**^**1**^	**Minimum ASIR**	**Maximum ASIR**
**Africa**			
Algeria, Sétif (1)	0.20	-	-
Egypt, Gharbiah (1)	0.30	-	-
Tunisia, Centre, Sousse (1)	0.20	-	-
Uganda, Kyadondo County (1)	1.30	-	-
Zimbabwe, Harare (1)	2.30	-	-
**America, Central and South**			
Argentina, Bahia Blanca (1)	1.30	-	-
Brazil (4)	2.98	2.50	4.50
Chile, Valdivia (1)	1.80	-	-
Colombia, Cali (1)	2.50	-	-
Costa Rica (1)	2.20	-	-
Ecuador, Quito (1)	1.80	-	-
La Martinique (1)	3.10	-	-
Peru, Trujillo (1)	3.20	-	-
**America, North**			
Canada^2^ (10)	1.40	0.9	1.8
USA^2^, National Program of Cancer Registries^3^ (35)	1.30	1.0	1.6
**Asia**			
Bahrain: Bahraini^4^ (1)	0.60	-	-
China (5)	1.17	0.20	1.60
India (6)	0.89	0.50	1.10
Israel (1)	0.80	-	-
Japan (7)	1.05	0.80	1.50
Korea^2^ (8)	1.40	1.20	2.20
Kuwait (2)	0.44	0.40	0.50
Malaysia (2)	2.63	2.20	2.90
Oman: Omani^4^ (1)	0.50	-	-
Pakistan, South Karachi (1)	0.80	-	-
Philippines, Manila (1)	2.20	-	-
Singapore (1)	2.00	-	-
Thailand (3)	3.44	2.40	3.90
Turkey (2)	0.62	0.60	0.70
**Europe**			
Austria^2^ (2)	0.90	1.30	1.30
Belarus (1)	0.90	-	-
Belgium (2)	1.06	0.90	1.10
Bulgaria (1)	1.30	-	-
Croatia (1)	0.90	-	-
Cyprus (1)	0.60	-	-
Czech Republic (1)	1.30	-	-
Denmark (1)	1.70	-	-
Estonia (1)	0.80	-	-
Finland (data not available)	-	-	-
France (11)	1.00	0.50	1.30
Germany (4)	1.05	0.90	1.30
Iceland (1)	1.60	-	-
Italy (22)	1.02	0.30	1.50
Latvia (1)	0.80	-	-
Lithuania (1)	1.40	-	-
Malta (1)	0.40	-	-
Norway (1)	1.60	-	-
Poland (2)	0.97	0.90	1.10
Portugal (2)	1.40	1.00	1.70
Russia, St Petersburg (1)	0.80	-	-
Serbia (1)	1.70	-	-
Slovak Republic (1)	1.40	-	-
Slovenia (1)	1.70	-	-
Spain (11)	1.10	0.50	1.70
Sweden (1)	1.60	-	-
Switzerland (7)	0.94	0.50	1.40
The Netherlands^2^ (2)	1.10	0.70	1.50
United Kingdom and Eire^5^ (12)	1.36	1.10	1.60
**Oceania**			
Australia (7)	1.18	1.00	1.30
French Polynesia (1)	1.20	-	-
New Zealand (1)	1.20	-	-

For countries with a single registry, the age-standardized incidence of cervical ADC for that registry is shown. For countries with multiple registries, the weighted average age-standardized incidence of ADC is shown. The number in parentheses after each country/city is the number of registries for that region. ^1^Data from Cancer Incidence in Five Continents (CI5) Volume IX (reference 26). ^2^Country level data provided by IARC; range from registry specific data (number of registries); Canada (10), Korea (8); Austria (2); Netherlands (2). ^3^National Program of Cancer Registries data used for USA country-level estimate; range from 35 state registries. ^4^Data provided for a race/ethnic group and not city. ^5^Includes data from 9 registries in England, 1 in Scotland, 1 in Northern Ireland and 1 in Eire.

**Figure 2:**
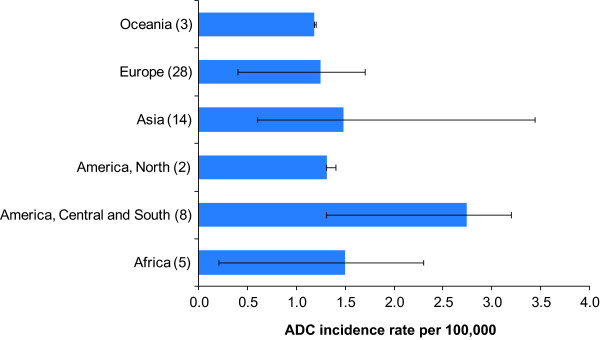
**Age-standardized incidence of cervical ADC by region.** Each bar represents the weighted average age-standardized incidence of cervical ADC for that region. Error bars show minimum and maximum values for individual countries within a region. Number in parentheses after region indicates number of countries contributing data. Incidence rates calculated using data from Cancer Incidence in Five Continents (CI5) Volume IX [reference 26].

Figure 2 groups the countries with available ASIR data [26] for ADC into world regions. The incidence of ADC was markedly highest in Central and South America (2.7 per 100,000 women), followed by Africa (1.5 per 100,000 women) and Asia (1.5 per 100,000 women). For comparison, the incidence of SCC in these same regions was 18.1 per 100,000 women in Central and South America, 22.9 per 100,000 women in Africa and 11.3 per 100,000 women in Asia.

### Effect of cervical screening on ADC

**Figure 3:**
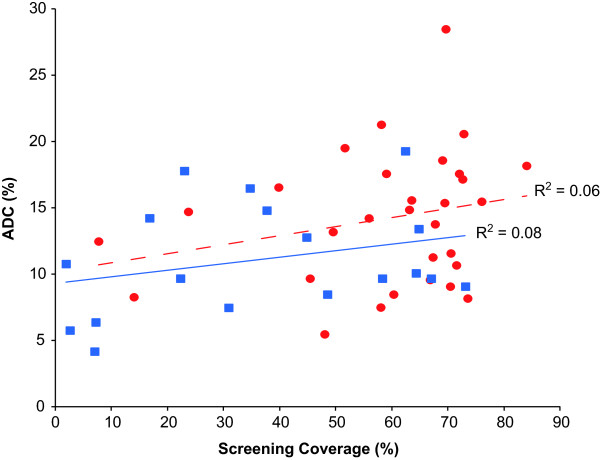
**Estimated percentage of cervical ADC versus estimated percentage of cervical screening coverage.** Red circle, developed countries; blue square, developing countries; red dashed line = trend line for developed countries; blue solid line = trend line for developing countries; R^2^, coefficient of determination. R^2^ for all countries (regardless of whether designated as developed or developing) = 0.13.

Information regarding cervical screening coverage was available from 48 countries (30 developed and 18 developing, as classified by IARC) of the 61 countries that had data reported for the proportion of ADC in ICC (see Figure 3). For these 48 countries (regardless of whether designated as developed or developing), there was a slight increasing trend in the percentage of ADC contributing to ICC with increasing cervical screening coverage (coefficient of determination [R^2^] = 0.13). This trend was not strong and was influenced by Finland (which has an estimated screening coverage of almost 70% and an estimated percentage ADC of 28.5%). When developed and developing countries were considered separately (Figure 3; red circle - developed, blue square - developing), there was a slight upward trend in percentage of ADC with increasing cervical screening coverage for both developed countries and developing countries, but the associations were weak (developed countries R^2^ = 0.06; developing countries R^2^ = 0.08).

### Type distribution of HPV in ADC

From the two published studies we investigated (Table 2), HPV DNA positivity in cases of ADC ranged from 65.6% [30] to 82.0% [29]. The most common HPV types in cervical ADC were HPV-16 (40.0 to 47.4%), HPV-18 (32.2 to 40.5%) and HPV-45 (5.7 to 11.8%). Based on the weighted averages from the two data sources, the proportion of all cervical ADCs associated with HPV-16 and/or 18 was approximately 80%. Three HPV types (HPV-16, -18 and -45) comprised approximately 87% of all cervical ADCs and five HPV types (HPV-16, -18, -45, -31 and -33) comprised approximately 92% of all cervical ADCs.

**Table 2:** Global HPV type distribution in cervical ADC (including ASC)

**HPV type**	**Li et al., 2011**^**1**^	**de Sanjosé et al., 2010**^**2**^	**Weighted average of both studies**
Number of cases	3525^3^	951	-
HPV positive cases, n (%)	2891 (82.0%)	624 (65.6%)	-
**HPV prevalence among HPV positive cases (%)**			
6	0.1	0.2	0.1
11	0.1	0.0	0.1
16	40.0	47.4	41.6
18	40.5	32.2	38.7
26	0.0	0.0	0.0
30	0.0	0.2	0.0
31	2.5	1.0	2.2
33	2.4	1.0	2.1
34	0.0	0.0	0.0
35	0.7	0.6	0.7
39	0.9	0.8	0.9
40	NT	0.0	-
42	NT	0.0	-
43	NT	0.0	-
44	NT	0.0	-
45	5.7	11.8	7.0
51	0.7	0.6	0.7
52	1.3	0.2	1.1
53	0.2	0.2	0.2
54	NT	0.0	-
56	0.2	0.3	0.2
58	1.7	0.5	1.4
59	0.9	0.8	0.9
66	0.1	0.2	0.1
67	0.2	0.0	0.2
68	0.6	0.3	0.5
69	0.9	0.0	0.7
70	0.2	0.0	0.2
73	0.0	0.0	0.0
74	NT	0.0	-
82	0.1	NT	-
85	0.0	NT	-
Undetermined	NR	1.3	-

ADC, adenocarcinoma; ASC, adenosquamous carcinoma; NR, not reported; NT, not tested. ^1^We adjusted prevalence rates from those reported by Li et al. [29] to indicate the HPV type-specific contribution to the total number of positive samples. In addition because women infected with multiple types contribute multiple times in the numerator but only once in the denominator, the addition of the total percentages exceeded 100; therefore we also adjusted these estimates to sum to 100%. ^2^de Sanjosé et al. [30] reported prevalence rates for ADC and ASC separately; we have added the estimates together to give an estimate of type-specific prevalence for ADC (including ASC). Prevalence rates indicate the HPV type-specific contribution to the total number of positive samples. Multiple infections were added to single types through proportional weighting attribution. ^3^HPV positivity data reported for only 3,525 of the 3,538 total cases of ADC.

### Potential impact of HPV vaccination

**Figure 4:**
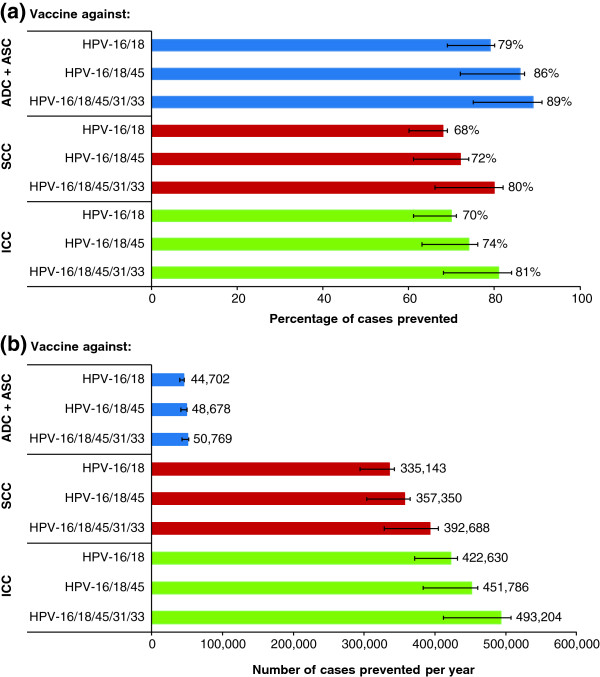
**Theoretical global impact of prophylactic HPV vaccination on cervical ADC, SCC and ICC.** Panel **(a)** and panel **(b)** show future predictions, using 2015 data, of the proportion of new cases and number of new cases, respectively, of ADC (including ASC), SCC and ICC which could theoretically be prevented globally by a HPV vaccine efficacious against HPV-16/18, HPV-16/18/45 or HPV-16/18/45/31/33. Each bar represents the point estimate. The numbers to the right of each bar are the actual point estimates. The error bars represent the corresponding range of estimates calculated using the lower and upper bound limits of the confidence intervals from reported efficacy estimates (96.1% and 95.89% confidence intervals were used for estimates of HPV-16/18 associated vaccine efficacy for *Cervarix*® and *Gardasil*®, respectively, due to adjustments for multiplicity. 95% confidence intervals were used for estimates of vaccine efficacy against other HPV types). We assumed 100% vaccine coverage in these calculations.

The potential impact of a prophylactic HPV vaccine assuming 100% vaccine coverage is shown in Figure 4. The preventative potential of an HPV vaccine is slightly greater for cervical ADC than for SCC: it was estimated that a vaccine which protects against infection with HPV-16 and -18 may theoretically prevent 79% (range of estimates calculated using the lower and upper bound limits of the confidence intervals from reported efficacy estimates: 69% to 80%) of new ADC cases and 68% (60 to 69%) of new SCC cases (Figure 4a). However, as the proportion of SCC cases relative to ADC cases is much larger, this translates into the theoretical prevention of 44,702 (39,228 to 45,614) cases of ADC and 335,143 (294,105 to 341,983) cases of SCC per year for a vaccine providing protection against HPV-16 and -18 (Figure 4b).

It is estimated that an additional 6,067 cases of ADC per year and 57,545 cases of SCC per year could be prevented due to cross-protective efficacy against HPV-31, -33 and -45. This would result in the theoretical prevention of 50,769 (41,931 to 42,356) cases of ADC and 392,688 (321,360 to 327,904) cases of SCC per year for a vaccine providing protection against HPV-16, -18 -45, -31 and -33 (Figure 4b).

## Discussion

The incidence of cervical ADC is rising and year-on-year comprises an increasingly larger proportion of all cervical cancer cases [3]. Using the latest available data, we estimated that ADC comprises almost a tenth of ICC globally and that the global burden of ADC is significant, reaching almost 60,000 new cases in 2015. Five HPV types (HPV-16, -18, -45, -31 and -33) together comprise over 90% of all cervical ADCs. Our data show that vaccines protective against HPV-16 and -18 could potentially prevent 44,702 cases (79%) of ADC globally per year, assuming 100% vaccine coverage. Vaccines with good cross-protective efficacy against other HPV types, including -45, -31 and -33, could potentially prevent an additional 6,067 cases (89%) of ADC globally per year.

We observed that the proportion and age-standardized rates of ADC were very variable between regions and countries, and even within countries. It is known that there is variation in the pathological diagnosis of histological types of ICC between diagnostic centres and diagnosis may be recorded in different ways in medical records. Other studies have noted wide intra-observation between colposcopists and pathologists in the ability to correctly identify morphologically diverse ADC [11,12]. ADC and SCC seem to share similar risk factors such as number of sexual partners, age at first sexual intercourse and use of oral contraceptive, but differ by parity, tobacco smoking and obesity [9,34-36] and it is likely that distribution of these risk factors differ by country/region. Variation in type-specific HPV prevalence may also be a contributing factor to the differences observed between countries [37]. Additionally, cervical screening may be more effective in detecting SCC than ADC [38]. There was some indication that regions with higher screening coverage may be associated with a higher proportion of ADC in ICC, however, our data were too limited to draw any firm conclusions as evidenced by the low correlation coefficients reported.

In the two data sources we selected, HPV DNA positivity in cases of ADC (including ASC) were 65.6% [30] and 82.0% [29], respectively. The relatively large proportion of HPV negative cases in the de Sanjosé study occurred despite the use of the highly sensitive SPF_10_ system for detection of HPV in formalin-fixed paraffin-embedded tissue and, according to the study authors, was likely due to technical artefacts, including tissue degradation (since this study reassessed samples archived as far back as 1949) and low viral load [30]. Li and colleagues did a meta-analysis of data from different studies published from 1990 to 2010 [29], which included a mix of fresh/fixed biopsies or exfoliated cells and prospective and retrospective testing using SPF_10_ and other less sensitive PCR based detection methods [39-41].

The low HPV detection rates may also be partly attributed to misdiagnosis of endometrial carcinoma as cervical ADC [42,43], but even in studies with careful histopathological review detection rates for ADC tend to range around 80-85% compared with 90-95% for SCC [44,45]. Unlike SCC which is generally acknowledged to be 100% attributable to HPV, some small proportion of rare histological variants of ADC (nonmucinous adenocarcinomas including clear cell, serous, and mesonephric carcinomas) likely arise independently of exposure to HPV [46,47]. The lower HPV detection rates for ADC versus SCC may be attributable to higher susceptibility of cervical ADC tissue to DNA degradation, due to lower DNA copy number for example, but this remains unclear and needs to be further investigated. There is also some evidence that HPV detection in cervical ADC may be improved by targeting of early protein E6 rather than the L1 segments that are targeted by SPF_10_[48,49].

If any falsely HPV DNA negative samples were attributable to a particular HPV type, the missing HPV types could theoretically bias the overall HPV type distribution [30]. Although this possibility cannot be ignored, the HPV type distribution in ADC (including ASC) was generally similar across both data sources, with HPV-16 and -18 being the most common types, followed by HPV-45 and then HPV-31 and -33 [29,30]. Any observed differences in HPV type distribution across the two data sources may be due to geographical variation, though it was not possible for us to quantify such differences as type-distribution data for ADC/ASC were not broken down by geographic region.

HPV types are traditionally grouped into phylogenetically-related species based on the genetic similarity of their L1 genes [50]. High-risk HPV types that cause cervical cancer fall largely, though not exclusively, within the *Alphapapillomavirus* 9 (alpha-9) species characterized by HPV-16 and including types -31, -33, -35, -52 and -58 and the alpha-7 species characterized by HPV-18 and including types -39, -45, -59 and -68 [51]. It is well documented that progression to SCC is greater for HPV-16 than HPV-18, but that HPV-18 is over-represented in ADC compared to SCC [52]. Both data sources used in our analysis confirm the limited genotype distribution among ADC, and the relatively high contribution of alpha-7 species such as HPV-18 and HPV-45. The greater tendency of alpha-7 species to cause ADC compared to alpha-9 and other species could be due to a phylogenetic trait, such as a greater tropism for infection of cervical glandular tissue or the multipotential cells of the squamocolumnar junction, and/or a better ability to neoplastically transform glandular cells once an infection is established [52,53].

The incidence of multiple infections amongst overall ICC was comparable in both data sources (7% in the meta-analysis [29] and 11.2% in the cross-sectional analysis [30]). Additionally, the incidence of multiple infections was similar between SCC and ADC (only the meta-analysis provided these data). The effect of multiple infections with respect to attribution to ICC and vaccine efficacy is unknown.

Prophylactic HPV vaccination has the potential to prevent a higher proportion of ADC cases than SCC cases, but the absolute number of ADC cases is still very small compared to the number of SCC cases. Our data show that vaccines protective against HPV-16, -18, -45, -31 and -33 could potentially prevent 89% of HPV-related ADC cases, accounting for 50,769 cases of ADC globally per year, assuming 100% vaccine coverage and a true HPV causal relation of 100%.

Our predictions do have some limitations and caveats. In estimating the potential impact of prophylactic HPV vaccines on cervical ADC, we assumed that 100% of ADC cases were due to HPV. However, we recognize that it is likely that although the vast majority (80% or more) [29] of routinely diagnosed cervical ADC are almost certainly caused by oncogenic HPV, a small proportion of such cases, especially those in the less frequent histological subtypes, are probably not associated with HPV [46,54,55] and we may, therefore, have overestimated the impact of HPV vaccination in preventing ADC. We did not specifically look at the role of multiple infections in ADC and the role of individual HPV types in causing cancer in multiple infections. We did not investigate the role of future changes in HPV infection rates. Additionally, as we wanted to investigate the potential benefit of HPV vaccination, we assumed that vaccine coverage was 100%, although we recognize that vaccine coverage may be much lower, particularly in less developed countries in which the burden of disease is highest.

## Conclusions

Prevention of cervical ADC through detection of preneoplastic stages has proved to be largely unsatisfactory, as observed by the high proportion of ADC in well-screened populations. We predict that the currently available HPV vaccines will be highly effective in preventing cervical HPV-related ADC. Extended protection against HPV-45, -31 and -33 would also be of benefit.

*Cervarix* is a registered trade mark of the GlaxoSmithKline group of companies.

*Gardasil* is a registered trade mark of Merck & Co Inc.

## Abbreviations

ADC: Adenocarcinoma; AIS: Adenocarcinoma *in situ*; ASC: Adenosquamous carcinoma; ASIR: Age-standardized incidence rate; CI5: Cancer Incidence in Five Continents; IARC: International Agency for Research on Cancer; ICC: Invasive cervical carcinoma; HPV: Human papillomavirus; R2: Coefficient of determination; SCC: Squamous cell carcinoma; WHO/ICO: World Health Organization/Institut Català d'Oncologia.

## Competing interests

All costs related to the development of this manuscript were met by GlaxoSmithKline Biologicals SA.

JMP is employed by GlaxoSmithKline Research and Development, and holds shares in GlaxoSmithKline; CG and SMT are employed by GlaxoSmithKline Vaccines, and hold shares in GlaxoSmithKline. DJ is a former employee of GlaxoSmithKline Vaccines and has in the past acted as a consultant to GlaxoSmithKline Vaccines. He has not received payment for work on this study.

## Authors’ contributions

JMP and DJ conceived the investigation. JMP and CG conducted the analyses. JMP, CG and SMT drafted the manuscript. DJ critically reviewed the manuscript and revised it for important intellectual content. All authors read and approved the final manuscript.

## Pre-publication history

The pre-publication history for this paper can be accessed here:

http://www.biomedcentral.com/1471-2407/13/553/prepub

## Novelty and impact

We estimate the burden of cervical adenocarcinoma (ADC) worldwide and the potential impact of human papillomavirus (HPV) vaccination against this disease. Our projections suggest that vaccines providing protection against HPV-16/18 may theoretically prevent 79% of new HPV-related ADC cases (44,702 cases annually) and vaccines additionally cross-protecting against HPV-31/33/45 may prevent 89% of new HPV-related ADC cases (50,769 cases annually). It is predicted that the currently available HPV vaccines will be highly effective in preventing cervical ADC.
